# Preliminary research on the identification system for anthracnose and powdery mildew of sandalwood leaf based on image processing

**DOI:** 10.1371/journal.pone.0181537

**Published:** 2017-07-27

**Authors:** Chunyan Wu, Xuefeng Wang

**Affiliations:** Research Institute of Forest Resource Information Techniques, Chinese Academy of Forestry, Beijing, China; Tallinn University of Technology, ESTONIA

## Abstract

This paper presents a survey on a system that uses digital image processing techniques to identify anthracnose and powdery mildew diseases of sandalwood from digital images. Our main objective is researching the most suitable identification technology for the anthracnose and powdery mildew diseases of the sandalwood leaf, which provides algorithmic support for the real-time machine judgment of the health status and disease level of sandalwood. We conducted real-time monitoring of Hainan sandalwood leaves with varying severity levels of anthracnose and powdery mildew beginning in March 2014. We used image segmentation, feature extraction and digital image classification and recognition technology to carry out a comparative experimental study for the image analysis of powdery mildew, anthracnose disease and healthy leaves in the field. Performing the actual test for a large number of diseased leaves pointed to three conclusions: (1) Distinguishing effects of BP (Back Propagation) neural network method, in all kinds of classical methods, for sandalwood leaf anthracnose and powdery mildew disease are relatively good; the size of the lesion areas were closest to the actual. (2) The differences between two diseases can be shown well by the shape feature, color feature and texture feature of the disease image. (3) Identifying and diagnosing the diseased leaves have ideal results by SVM, which is based on radial basis kernel function. The identification rate of the anthracnose and healthy leaves was 92% respectively, and that of powdery mildew was 84%. Disease identification technology lays the foundation for remote monitoring disease diagnosis, preparing for remote transmission of the disease images, which is a very good guide and reference for further research of the disease identification and diagnosis system in sandalwood and other species of trees.

## 1 Introduction

Digital image processing technology has been widely used in the field of agriculture [1,2], and also can closely monitor the diseases that affect plant growth [3], identify and diagnose plant leaf diseases, capture the core content for remote exploration of multi-spectrum and high-spectrum sensing images [4]. Using digital image processing and analysis technology for plant disease detection and recognition has become one of the important means for disease diagnosis with the improvement of computer processing ability and the continuous development of digital image acquisition devices [5,6]. The BP (Back Propagation) neural network is one of the most widely used neural network models currently [7]. Using the BP neural network for image segmentation, recognition, recovery, matching and classification analysis. We also applied this technology to the forest models research area. The BP neural network is applied to a segment of the sandalwood anthracnose lesion based on that [8]. The SVM is a kind of pattern recognition method based on statistical learning theory, which shows many unique advantages in solving small sample, nonlinear and high-dimensional pattern recognition problems [9]. As a result, some applications of the SVM method have been reported in the field of plant disease image recognition. Most of the previously available imaging techniques for disease diagnosis are based on naive Bayes classification algorithm or Neural Network method with simply shape extraction. While SVM technology has good generalization ability, high dimensional processing ability and strong nonlinear processing ability that the previous image technology have not these advantages. Yang [10] compared to the naive Bayes classification algorithm, BP network and SVM for image classification showing that SVM have better classification ability than the other two classifiers whether using color information or texture information.

Sasaki et al. [11] researched the image automatic diagnosis technology for anthracnose in cucumbers in 1999, and developed the relevant model for analysis of cucumber disease images in 2003, Sammany et al. [12] integrated the neural network and SVM technology to improve the accuracy of image recognition in 2006. In China, the research on the identification of crop diseases and insect pests started relatively late than abroad. In 2001, Mao et al. [13] began studying the image recognition of leaf nutrient deficiency and extracted the color and texture characteristics of tomato leaves with nutrient deficiency. From 2004 to 2007, much research on the application of the SVM method in disease and insect pest recognition was conducted by Tian et al. [14], which discovered that the SVM method has high recognition performance. *Santalum album Linn*, a semi-parasitic small tree of the genus *Santalum* and of the family *Santalaceae*, is one of the most expensive types of wood [14,15,16,17,18] and is listed as one of the national key protected plants. Sandalwood have long medical history [19], based on the object of research, the pathogen of seedlings early destruction [20], the articles about its spike disease [21], and the spike disease phytoplasma identification [22,23], and report about sandalwood ulcer disease problems. Liu [24] showed that the main diseases of sandalwood were powdery mildew, anthracnose, sooty blotch, seedling blight and canker. The harm degree of anthracnose and powdery mildew in Hainan province is gradually becoming serious, especially for young sandalwood in recent years. However, at present, there are a limited number of studies on sandalwood disease at home and abroad.

Anthracnose caused by *Colletotrichum* or *Gloeosporium*, it mainly occurs in the plant leaves, its often damage leaf margin and tip, serious, most part of leaf becoming black and the dead. Produce round or nearly round lesion in the stem, there is a black dot in the pattern with pale brown. Lesion occurred on the tender shoots with oval ulcer plaque, margin slightly uplift. Sandalwood blight disease caused by anthracnose and harms the tender leaves, flowers, fruit and shoot of sandalwood. It mainly harms the tender leaves and fruit of sandalwood in the Hainan area. The re-infection ability of the disease is strong, it will spread rapidly when the condition is met. Leaves will appear purple or will have purple-brown spots in the early stage of the disease [25], it might lead to the defoliation of many leaves ahead of schedule when disease becomes serious, which seriously influences the growth of the sandalwood.

Powdery mildew caused by pathogens causing, specificity parasitic on the surface of plants and produce pathogenic fungi with white powder disease symptoms. They belong to ascomycotina pyrenomycetes Erysiphales erysiphaceae, having higher parasitic specificity. It mainly occurs in plants at Seedling Stage. it began to produce yellow dots on the leaves, and then, expanded into a round or oval spots with white powdery mildew layer on the surface. In general, the lower part of the blade is more than the upper leaves, and the back of the blade is more than the front. Mildew disperses individually in the early stage, and unites into a large blotch later, which can even cover the whole leaf. Photosynthesis can be seriously affected, the normal metabolism is disrupted resulting in premature aging and production loss [24,26,27,28,29]. Currently, there has not yet been any systematic study on the anthracnose and powdery mildew of sandalwood.

Therefore, in 2014–2016, we observed the characteristics of anthracnose and powdery mildew disease for sandalwood in the Hainan provincial state-owned ‘Daodong’ forest farm. We segmented the image lesion and extracted related features of the sandalwood anthracnose and powdery mildew by using image processing technology, with the feature parameter vector as the input of support vector machine. The classification research on the two kinds of disease for sandalwood provides the basis for diagnosis of sandalwood anthracnose quickly and accurately. This research is of much importance to the health management of the sandalwood in the Hainan province.

The authority responsible for a national park is the Hainan provincial state-owned ‘Daodong’ forest farm. That has no specific permissions were required for the location, and confirm that the field studies did not involve endangered or protected species.

## 2 Materials and methods

### 2.1 Site conditions

Choosing the Hainan provincial state-owned ‘Daodong’ forest farm as main research area, which is located in the territory of Wenchang, in the northeast of Hainan at 110°36′~111°01′E, 19°40′~20°06′N, the total land area is approximately 12 thousand hm^2^. The forest farm is on the coastal plain, the altitude is 5 to 20 m in most areas. The landscape is flat and wide. The survey ground is a tropical marine monsoon climate. In particular, an annual mean temperature is 23.9°C, the lowest temperature is 0.3°C in January, an annual average accumulated temperature more than 10°C is 8474.3°C, a total solar radiation energy is 453.3~479.1 kJ·cm^-1^, abundant rainfall with uneven distribution, typical dry and wet seasons, a serious spring drought, an annual precipitation is 1721.6 mm. The vegetation type in this area is a tropical monsoon rainforest; the main operating tree species are *Casuarina equisetifolia*, *Pinus elliottii*, *Eucalyptus robusta*, *Acacia mangium*, *Acacia auriculiformis*, and *Acacia crassicarpa*; the total forest area is more than 20 million acres. However, the high temperatures and rainy weather in Hainan have provided a suitable living environment for the breeding and spreading of diseases and insect pests, which have adverse effects on the development of forestry production.

### 2.2 Experimental materials

The field monitoring system of sandalwood is established as a servo instrument remote monitoring site for sandalwood images in the Hainan provincial state-owned ‘Daodong’ forest farm. We use sensors and embedded hardware technology monitor growth conditions and disaster characteristics of sandalwood. It can also receive field plant images in real-time by an HD controlled camera. The data transmission, remote control and management functions were realized by wireless network. It operates on-site monitoring and data analysis intelligent application in sandalwood.

This time, we obtained anthracnose and powdery mildew images of sandalwood leaves by Real-time monitoring platform, and screen and identify the leaf image quality by the brightness and clarity of image, and extract the disease leaf images of sandalwood with moderate resolution and brightness. Research on disease diagnosis with images acquired from the field lays the foundation for remote monitoring a large number of sandalwood diseases.

### 2.3 Image recognition model design

The specific principles and methods of image recognition model include image segmentation, image feature extraction and SVM recognition. The program is realized by language programming through the MATLAB 7.11 platform.

#### 2.3.1 Disease image segmentation method

***Input layer***: This layer is used for the data input of the system, and it is represented by an input vector U = (u_1_, u_2_, …u_n_)^T^. We take the color features of the images as segment features. We take n = 9, that is the RGB values and the gray value of the 8 adjacent points around the pixel points in the image of the input sample image, which we call N_r_, which is composed of an input mode considered as 9-dimensional vectors:

$$I=\{R,G,B,N_{r1},N_{r2}\cdots ,N_{r8}\}$$ (1)

***Hidden laye****r*: This layer is the input component considered as an n-tuple (u_1_, u_2_…u_n_) based on a certain non-distinguishable relation, which determines the link between each entry and their respective categories. Each input component is discretized into different values of “ri” between 0 and 1.

***Output layer***: The layer is composed of q nodes representing the output variables. In this paper, q = 2, the output foreground is 0, and the background is 1 [30].

***This paper algorithm***: We analyzed the image, determined the range of the foreground and background colors, and then stored the foreground and background colors in an array according to the order. This array is the training sample array. In addition, then, we set up an array with the same size to preserve the characteristic value of the sample. The value is 1 for the foreground and 0 for the background.

We put the sample value and characteristic value into the BP neural network. The BP neural network, at first, searches for a set of the most appropriate weights and thresholds in the set threshold value space, and then, sets those values into the initial threshold values of the neural network. Then, the training is carried out until the mean square error converges to a specified value or the maximum number of iterations is reached. The neural network is optimal with respect to time.

Image segmentation can be viewed as a process of classification. Each pixel (D_ij_) in the image (D) is a sample to be classified; this sample is sent into the BP neural network (SIM) for classification, and it outputs a characteristic value V_i_, which determines the probability that the sample belongs to a class. We use the convention that if the value is greater than 0.5, it is in the foreground (F); otherwise, it is in the background (B) [31].

$$V_{ij}=sim\left(D_{ij}\right)$$ (2)

$$S_{ij}=\{\begin{array}{l} F,V_{ij}>0.5\\ B,V_{ij}<0.5 \end{array}$$ (3)

#### 2.3.2 Disease image feature extraction

***Shape feature extraction***: Shape information having invariability in displacement, rotation and scale transformations is an image stability feature. After lesion extraction, we extract the area feature information by image shape difference, which can directly reflect the characteristics of different diseases [32].

***Color feature extraction***: Using the RGB color space to analyze the lesion color, we extract the RGB color components of the suspected lesion, and use B/G and R/G to standardize the color characteristics. A total of five color feature vectors were obtained [33].

***Texture feature extraction***: The gray level co-occurrence matrix is defined from the image gray level of K (where the position is (i, j)), and the distance from K is d, the direction is *θ*, the probability of simultaneous emergence is *p*(*k*, *l*, *d*,*θ*), and the pixels with gray level of l (when the position is (*i+d*_*i*_, *j+d*_*j*_)). This is expressed mathematically by the following formula:

$$p\left(k,l,d,\theta \right)=\left\{\left[\left(i,j\right),\left(i+d_{i},j+d_{j}\right)|f\left(i,j\right)=k,f\left(i+d_{i},j+d_{j}\right)=l\right]\right\}$$ (4)

Where (i, j) is the coordinate of the pixel; k, l are the gray values; d_i_, d_j_ is the position offset; d is the distance between the two pixels; and *θ* is the generation direction of the gray level co-occurrence matrix. When there are L gray levels, Haralick et al. defined the characteristics of the 14 gray level co-occurrence matrix for texture analysis. In this paper, we use the following four parameters as the texture characteristic value of the disease.

***Energy***:

$$ASM=\sum_{i=0}^{L-1}\sum_{j=0}^{L-1}p{\left(i,j\right)}^{2}$$ (5)

Energy is a measurement of the uniformity of the image intensity distribution. The ASM value is large when the texture is coarse, and the ASM value is small when the texture is fine.

***Contrast***:

$$CON=\sum_{i=0}^{L-1}\sum_{j=0}^{L-1}{\left(i-j\right)}^{2}p{\left(i,j\right)}^{2}$$ (6)

The contrast reflects the clarity of the image texture. The CON value is large with deep grooves texture, the effect is clear, the CON value is small with a shallow groove texture, and the effect is fuzzy.

***Entropy***:

$$ENT=-\sum_{i=0}^{L-1}\sum_{j=0}^{L-1}p(i,j)\text{log }p(i,j)$$ (7)

Entropy is a measurement of the amount of information in the image. The ENT value is large when the texture is complex, the ENT value is small when the texture is simple, and the ENT value is almost 0 when the image does not have any texture.

***Correlation***:

$$COR=\frac{\sum_{i=0}^{L-1}\sum_{j=0}^{L-1}ijp(i,j)-u_{1}u_{2}}{\sigma_{1}^{2}\sigma_{2}^{2}}$$ (8)

Where $u_{1}=\sum_{i=0}^{L-1}i\sum_{j=0}^{L-1}p(i,j)$, $u_{2}=\sum_{i=0}^{L-1}j\sum_{j=0}^{L-1}p(i,j)$, $\sigma_{1}^{2}=\sum_{i=0}^{L-1}{\left(i-u_{1}\right)}^{2}\sum_{l=0}^{L-1}p\left(i,j\right)$, and $\sigma_{2}^{2}=\sum_{i=0}^{L-1}{\left(i-u_{2}\right)}^{2}\sum_{j=0}^{L-1}p\left(i,j\right)$.

Correlation corresponds to the similarity degree of the elements of the gray level co-occurrence matrix in the direction of the row or column.

We calculated the energy, contrast, entropy, correlation parameters in four directions: 0°、45°、90°、and 135°. The mean and variance of the energy, contrast, entropy, and correlation are eight texture feature parameters.

#### 2.3.3 Support vector machine classification design

***SVM basic principle***: The SVM transforms the input space into a high-dimensional space by a nonlinear mapping, and then in this new space, it obtains the optimal separating hyper plane of the maximum interval sample classification, defined by the appropriate inner product function (kernel) achieve the nonlinear transform. The calculation method of the discriminant function is as follows [34,35,36]:

$$f\left(x\right)=\text{sgn}(\overset{N}{\underset{k=1}{\sum }}y_{k}\times \alpha_{k}^{*}\times Q\left(x,x_{k}\right)+b^{*})$$ (9)

Where f is a classification function, sgn is a symbolic function, {(x_k_, y_k_), k = 1, 2, 3, ….., N} is a sample set, N is the sample size, x is the input feature vector, y is the assigned category, Q(x, x_k_) is a kernel function, and b* is the threshold of classification.

We express the displacement of the optimal classification plane, which is optimal coefficient vector and is also a Lagrange multiplier.

The kernel function design of the SVM is very important, and it is related to the classification result. Because linear kernel function relatively simple, its only suitable for linear separable problems. However, this limitation is big, nonlinear classifiers have wide applicability, their analyses and calculations are complex. Statistical pattern recognition is one of the methods used to SVM classification in digital image recognition, it can make a scientific judgment based on information value.

Commonly used SVM kernel function include:

***Linear kernel functions***:

Q(x,y)=x⋅y (10)

***Polynomial kernel functions***:

$$Q(x,y)={\left[(x\cdot y)+1\right]}^{d}$$ (11)

***Radial basis kernel functions***:

$$Q(x,y)=\text{exp}-\frac{{\left(x-y\right)}^{2}}{\sigma^{2}}$$ (12)

***Sigmoid kernel functions***:

Q(x,y)=tanh[Q(x⋅y)+t] (13)

A large number of studies show that using SVM with different kernel functions can classify the disease leaf image samples of sandalwood, and the classification performance of the radial basis kernel function is the best and stable [37]. Therefore, in this paper, the radial basis kernel function was selected as the optimal function in finally testing and improving the above functions. Finally, we get the best function for this paper by improving on that basic formula:

$$Q(x,y)=\text{exp}-\frac{\left|x-y\right|}{2\sigma^{2}}$$ (14)

***SVM classifier design***:

The feature parameter vector data was normalized to the range [0, 1].

To realize the transformation from high dimension to low dimension and transform the original feature combination into a new feature combination, the dimension of the feature parameter vector was reduced.

The SVM parameters were optimized to get the best recognition rate.

The samples were trained, classified, tested and classified again after processing the data and selecting the parameters by the above features.

To select the best parameter combination and obtain the optimal recognition rate, we use a stepwise discrimination algorithm test on a contribution of the selected feature parameters.

Based on this, we use the color, texture and shape disease feature vector that is extracted from the image and segmented based on lesion location in this study. The disease diagnosis model was established based on the radial basis kernel function, and then, we input categorizing images into the vector machine, which were classified and predicted by the trained model.

#### 2.3.4 Disease diagnosis

We processed the 200 images selecting from the database by the image recognition model. We took 150 (each of anthracnose, powdery mildew and healthy leaves were 50) of the above-mentioned 200 samples as the training set, and the other 50 as the test set.

We inputted 10 feature vectors of anthracnose, powdery mildew and health samples into the vector machine after preprocessing. Lesion segmentation and feature extraction were performed for each image, and then, the radial basis kernel function was used to distinguish the disease. To select the optimal combination of feature parameters, the stepwise discrimination algorithm test was used on the contribution of each characteristic parameter, and the image disease diagnosis of sandalwood anthracnose and powdery mildew was performed, and then we obtained the optimal diagnosis rate.

## 3 Results

### 3.1 Disease image segmentation results

After performed statistical analysis all the data and image processing, we obtained a better result in disease leaf image segmentation by the BP neural network. Among the results, the black spot extracted from the leaf image is the site of the anthracnose disease, while the white spot extracted from the leaf image is the site of the powdery mildew. Select part of the image as a representative to display the results. S1 Fig. shows parts of the segmented images.

### 3.2 Disease image feature extraction results

We segment the suspected lesion for the all samples using the disease plaque segmentation method proposing in this paper. In addition, we extracted the color (R, G, B, R/G, B/G), texture (energy, entropy, contrast, correlation) and shape (area) 10 feature vectors of the segmented image, after statistical analysis of the data, the result as shown in S1 Table. Texture features extracted from the images are shown in S1(C) Fig. The features of different diseases are different, the distinguishing degree being more obvious. Because powdery mildew showed a white lesion, the mean values of R/G and B/G are all in the vicinity of 1, while the B/G component of the anthracnose and healthy leaves samples is significantly lower than 1. The suspected lesion area of healthy leaves is far smaller than that of powdery mildew and anthracnose samples. Therefore, the area is an important characteristic parameter for healthy sample identification. However, the dispersion degree of the disease characteristic value is large and has poor stability. This is particularly evident of the feature vector in the area, where the standard deviation is significantly larger than that of the other vectors.

S2 Fig. shows the distribution of 10 characteristics for 150 samples, where the blue diamonds represent powdery mildew, the red squares represent the anthracnose, and the green triangles represent a healthy sample. Differences in color feature of diseases and insect pests were more obvious than the texture and shape features, and the R/G and B/G components had a certain ability to distinguish between the disease and the powdery mildew. Texture features and area features are helpful to distinguish healthy leaves from the unhealthy ones. Through the following scatter plots, we can fully distinguish the samples of the two diseases.

### 3.3 Support vector machine recognition results

#### 3.3.1 Test sample disease diagnosis

After analyzing and processing the data, the results show that 89 of 100 samples having two target types have been accurately classified based on the SVM with the radial basis function kernel, when the penalty parameter c = 12 and the kernel function parameter g = 0.0791. The classification accuracy of the training set is the highest, and the overall accuracy rate of recognition is 89%. This shows that the method used in this research have high accuracy and reliability, and it is feasible and promising for real-time diagnosis of diseases.

#### 3.3.2 Test sample breakdown diagnosis results

Results are shown in S2 Table, we took further statistics forthe tested samples diagnostic results. The correct identification rates of powdery mildew, anthracnose and healthy samples were 84%, 92% and 92%, respectively. Among them, two of them were wrongly identified as healthy leaves images, four images of powdery mildew were judged to be healthy leaf images and two images of healthy leaf image were thought to be of leaves with anthracnose. It is difficult to completely avoid errors in analysis of the sample, and in the results of this study, the identification accuracy of anthracnose and healthy samples were more than 90%. The identification accuracy of powdery mildew is also more than 80%, which is more difficult to identify. This shows that higher recognition accuracy can be obtained by using the SVM model, which can be applied to identify plant diseases and insect pests and provide support for disease prevention and management of sandalwood.

## 4 Discussions

Plant diseases are becoming more and more serious to the forest with climate change [38]. In this study, suspected lesion segmentation was carried out on anthracnose, powdery mildew and healthy leaf images in sandalwood. After that, the images having been segmented were feature extracted and SVM recognized, we obtained an ideal result. This provides the basis for an application of image recognition to the field of forestry.

Sandalwood disease diagnosis system is a promotion of forestry informatization being based on the BP neural network and SVM. It has made a new breakthrough for exploring the auxiliary methods of disease prevention and treatment in sandalwood. Relevant experts show that texture-related features might be used as discriminators when the target images do not follow a well-defined color or shape domain pattern, and feature-based image classification has many advantages [39,40,41]. Simultaneously, disease image recognition technology is more practical and intelligent due to the support of the BP neural network and SVM technology, which brings a newly developing direction to plant disease identification technology in the field of forestry. It is a good guide and reference for further study of disease recognition and diagnosis in sandalwood and other forest species.

Forestry has become more and more informative and intelligent with the rapid development of modern technology, the traditional forestry management mode will be replaced by modern forestry management with all kinds of high and new technology. This research will contribute to the development of modernization for promoting the study in sandalwood disease.

Artificial neural networks and support vector machines are commonly used in recognition models to identify plant diseases. Because the artificial neural network needs a large number of samples to be trained to obtain better results, and the experimental study of disease images was small, the support vector machine was chosen for the sandalwood disease recognition model [36]. Because light has a great influence on the feature parameters of color and has little influence on texture feature parameters, there is a higher level of accuracy for sandalwood disease recognition with both a color feature and a texture feature. In view of the color feature for sandalwood powdery mildew, segmentation for powdery mildew is more difficult than for anthracnose disease. It is particularly difficult to distinguish in the seedling stage of sandalwood leaves.

The study demonstrates that the results accuracy was improved when using the BP neural network algorithm to segment the diseased leaves images, and the method can effectively utilize the large scale parallel processing and greatly reduce the processing time. Good results can be obtained by using the BP neural network algorithm proposed in this paper to segment and extract the diseased leaves in sandalwood. The texture feature and color feature of images can be used as the feature vector to identify the disease image [42,43,44]. By extracting the shape feature, color feature and texture feature of the diseased images and by recognizing and classifying the diseases with the SVM, the results show that the correct rate of recognition and diagnosis system in this paper are above 80% and 90%, which shows that the diagnosis system can be used for disease diagnosis in sandalwood and that it can correctly identify the disease type of a field disease by using monitoring images in sandalwood.

This research is a preliminary exploration for image segmentation and recognition technology applications in the diagnosis of sandalwood. At present, only field shooting identification and diagnosis for anthracnose and powdery mildew disease of sandalwood have been studied. It is necessary for further research work on remote automatic recognition and judgment of sandalwood pest diagnosis and making the whole system more perfect.

The combination of disease identification technology and digital image processing technology will more conducive to improve the disease diagnosis system. At present, the recognition system uses only image information. It has some limitations, and carrying out a multi-source data fusion research is the next step. For example, we can study the relationship between meteorological conditions and the occurrence and development of diseases.

## Supporting information

S1 Fig The suspected lesion segmentation and feature extraction of three kinds of disease leaf with anthracnose, powdery mildew and health ones. Legend: A. Blade foreground image; B. Extraction image of lesion area by BP; C. Texture feature extraction image.
(TIF)

S2 Fig Images features distribution of leaves. Legend: (a) R value of three kinds of disease; (b): G value of three kinds of disease; (c): B value of three kinds of disease; (d): R/G value of three kinds of disease; (e): B/G value of three kinds of disease; (f): Energy value of three kinds of disease; (g): Entropy value of three kinds of disease; (h): Contrast ratio value of three kinds of disease; (i): Relevance value of three kinds of disease; (j): Area value of three kinds of disease.
(TIF)

S1 Table Image features of sandalwood leaves. (DOC)

S2 Table Recognition accuracy of the test sample with anthracnose, powdery mildew and healthy. (DOC)
