# Supplementary figures and images for: Preliminary research on the identification system for anthracnose and powdery mildew of sandalwood leaf based on image processing
Source: PLoS One. 2017 Jul 27;12(7):e0181537. doi: 10.1371/journal.pone.0181537 (PMC5531471; doi:10.1371/journal.pone.0181537)

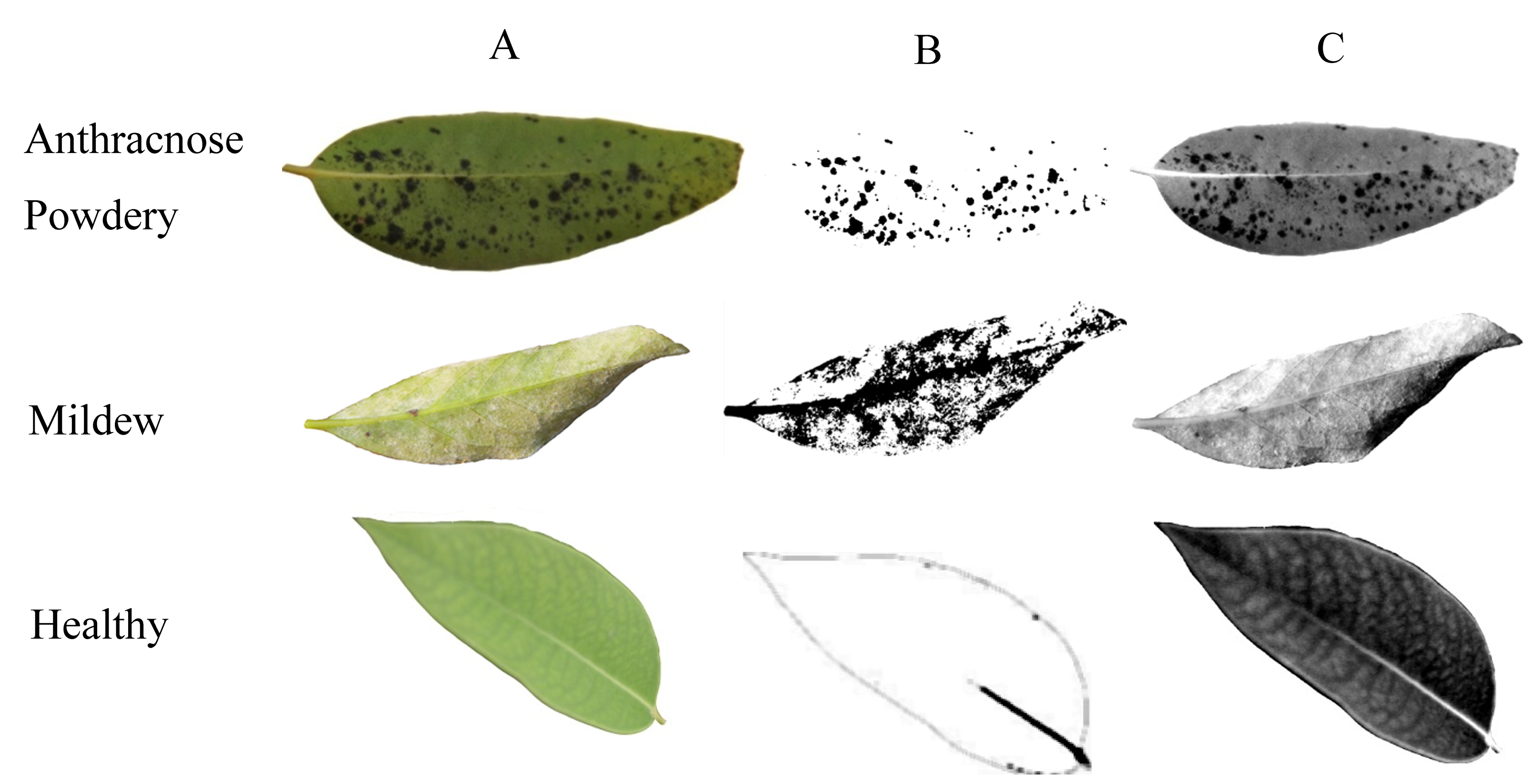

Supplement: S1 Fig — Legend: A. Blade foreground image; B. Extraction image of lesion area by BP; C. Texture feature extraction image. (TIF) [file pone.0181537.s001.tif]

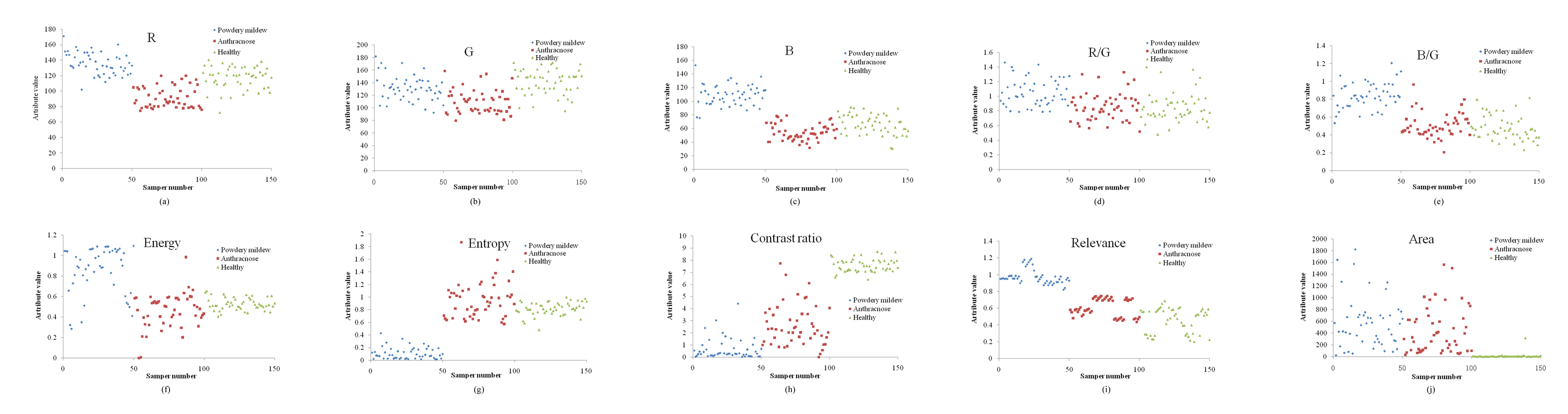

Supplement: S2 Fig — Legend: (a) R value of three kinds of disease; (b): G value of three kinds of disease; (c): B value of three kinds of disease; (d): R/G value of three kinds of disease; (e): B/G value of three kinds of disease; (f): Energy value of three kinds of disease; (g): Entropy value of three kinds of disease; (h): Contrast ratio value of three kinds of disease; (i): Relevance value of three kinds of disease; (j): Area value of three kinds of disease. (TIF) [file pone.0181537.s002.tif]
