# Supplementary material for: Preliminary research on the identification system for anthracnose and powdery mildew of sandalwood leaf based on image processing
Source: PLoS One. 2017 Jul 27;12(7):e0181537. doi: 10.1371/journal.pone.0181537 (PMC5531471; doi:10.1371/journal.pone.0181537)
Supplement: S1 Table — (DOC) [file pone.0181537.s003.doc]

**S1 Table.**

**Table** 1 Image features of sandalwood leaves

| **Character** | **Anthracnose** | | **Powdery mildew** | | **Healthy** | |
| --- | --- | --- | --- | --- | --- | --- |
|  | **Mean** | **Standard deviation** | **Mean** | **Standard deviation** | **Mean** | **Standard deviation** |
| **Red constituent** | 91.7792 | 13.4130 | 135.8242 | 14. 0230 | 117.4993 | 14.4272 |
| **Green constituent** | 109.7945 | 18.8131 | 131.3982 | 19.6739 | 141.4498 | 19.4903 |
| **Blue constituent** | 54.2803 | 11.7937 | 108.8687 | 15.1705 | 66.3187 | 14.7207 |
| **Red/green constituent** | 0.8590 | 0.1902 | 1.0540 | 0.1805 | 0.8536 | 0.1989 |
| **Blue/green constituent** | 0.5831 | 0.1354 | 0.8415 | 0.1405 | 0.4788 | 0.1352 |
| **Energy** | 0.9528 | 0.2736 | 0.1090 | 0.0943 | 0.8201 | 0.1057 |
| **Entropy** | 0.8201 | 0.1057 | 0.0574 | 0.0304 | 0.9528 | 0.2736 |
| **Contrast** | 3.7948 | 1.7256 | 0.4778 | 0.2007 | 4.8143 | 3.5766 |
| **Correlation** | 0.6018 | 0.1055 | 1.0948 | 0.2847 | 0.4567 | 0.1444 |
| **Area** | 436.2388 | 386.3587 | 571.3753 | 424.7732 | 12.5448 | 44.0933 |
