# Supplementary material for: Preliminary research on the identification system for anthracnose and powdery mildew of sandalwood leaf based on image processing
Source: PLoS One. 2017 Jul 27;12(7):e0181537. doi: 10.1371/journal.pone.0181537 (PMC5531471; doi:10.1371/journal.pone.0181537)
Supplement: S2 Table — (DOC) [file pone.0181537.s004.doc]

**S2 Table.**

**Table 2 Recognition accuracy of the test sample with anthracnose, powdery mildew and healthy**

| **Disease** | **No. of training sample** | **No. of texting sample** | **Anthracnose** | **Powdery mildew** | **Healthy** | **Correctness** |
| --- | --- | --- | --- | --- | --- | --- |
| **Anthracnose** | 50 | 25 | 23 | 0 | 2 | 92% |
| **Powdery mildew** | 50 | 25 | 0 | 21 | 4 | 84% |
| **Healthy** | 50 | 25 | 2 | 0 | 23 | 92% |
